# 2318: “A clash of cultures”: Cervical cancer screening and Hispanic males

**DOI:** 10.1017/cts.2017.259

**Published:** 2018-05-10

**Authors:** Bertha E. Flores, Martha Martinez, Lyda Arevalo-Flechas, Darpan Patel, Merlin Tobar, Deborah Parra-Medina

## Abstract

OBJECTIVES/SPECIFIC AIMS: Focus groups are being conducted to describe and identify barriers and/or facilitators to Hispanic males’ health literacy, culture, and language related to cervical cancer prevention practices METHODS/STUDY POPULATION: A purposive convenience sample was recruited to participate in focus group sessions with English or Spanish speaking Hispanic males 21 years of age and older. Groups were segmented by age (21–29, 30–39, 40–49, and 50–65), and language (English or Spanish). Focus group discussions (n=8) were led by a bilingual/bicultural female researcher using a discussion guide that followed Zarcadoolas *et al*. (2005) health literacy model 6 as related to their partners’ cervical cancer screening and prevention practices. All sessions were audio-recorded and transcribed verbatim. Participants completed standardized questioners regarding demographic data and their health literacy. Qualitative content analysis was used for analyzing focus group interviews. RESULTS/ANTICIPATED RESULTS: Preliminary qualitative analysis shows the struggle Hispanic males’ face accepting cervical cancer screening for their female partners. One participant reported that it was “a clash of cultures.” A “clash of cultures” was described as a constant struggle and acceptance between science, personal knowledge, and Hispanic cultural taboos. DISCUSSION/SIGNIFICANCE OF IMPACT: Hispanic male’s health literacy, communication, language preferences, and cervical cancer risks, will further enhance the knowledge needed to design intervention measures for cancer prevention among Hispanics. Understanding the factors that contribute to the unequal burden of cervical cancer incidence and mortality among Hispanic women in South Texas is critical to prevent cervical cancer among this population.

